# Answer to an Uncandid Review

**Published:** 1817-02

**Authors:** G. D. Yeats


*For the London Medical and Physical Journal.*

*Answer to an uncandid Review;* by G. D. YEATS, M.D.

I REQUEST the favour of a page or two of your Journal, as the medium for a few observations addressed to the Editor of the Critical Review published in October last.— The ingenious Editor has been good enough to take the trouble to notice my pamphlet on Water in the Brain; but, in so doing, has attached to me an opinion which I certainly do not entertain, and which I do not find (by a reference to the quoted paragraph from the pamphlet) the expressions entirely warrant. It is this—After having insisted, with much earnestness, on the indispensable necessity of attending, with minute care, to the earliest symptoms, which too frequently terminate in hydrencephalus, I have added,—"the necessity of such watchful attention is more indispensable in a curative point of view, on the subject of the present letter, in as much as, with all other diseases in which our art is at all available, a cure may be effected in almost any of the subsequent stages." The inference which the learned critic has drawn, does not appear to me to be quite logical, and certainly is very foreign from my intention to draw, viz. that the *obsta principiis* was not of great importance in other

---

the diseases. The correct inference is, that I think it of more importance in this than in most other diseases. I cannot avoid being of the same opinion still. The maxim *venienti occurrite morbo* is in every tyro's mouth, and should not be expunged from the recollection, nor excluded from the practice of the experienced physician. Experience, indeed, will teach him how very difficult it is to break the links of diseased actions, when the chain has been fully formed and continued in activity by habit, or has terminated in disorganized structure.

In hydrencephalus, the derangement in the functions of the digestive organs continues more or less long in different patients, previous to the affection of the brain: in some, the translation of the morbid action to the brain is so rapid that the child has slipped into a fatal disease before its friends are aware of the approach. With such a fact before me, and which I have many times witnessed, I do not think I could too impressively fix upon the minds of parents a steady attention to the very first deviations from health in their infant offspring. I therefore submit it to the ingenious Editor whether I was not justified in making the observations I did, as quoted above in the inverted commas.

I beg the Editor will receive these observations as explanatory of my meaning, and that it is likely, from the face of the expressions, I might afford an opportunity for just criticism, from the consideration that my attention has been fixed in watching the symptoms of this disease for many years. I have read, with much interest and satisfaction, more particularly the concluding part of the criticism. I consider it a matter of very considerable importance, which ought never to be lost sight of in practice, that local inflammation will not always be destroyed by general bleeding, however largely or often repeated; and even sometimes the local action is such as to baffle the effects of local depletion, however judiciously employed. I use the words *local action* as the terms usually resorted to, without wishing to enter into any theoretical discussion whether local inflammation arise from activity or torpor, although I incline to the opinion that activity of vessels is the cause. I take this opportunity of mentioning, that, in cases of local inflammation, of whatever part, where both local and general bleeding shall have failed to reduce the disease, the action still going on to wear out the patient, and further depletion becomes doubtful, if not dangerous, to the debilitated patient, I have, for many years, adopted a mode of practice which has carried with it a marked success. This is not the place for entering upon the subject, which may probably be the em-

 ployment ployment of some future day. I may, however, add, that the subject has been mentioned with some very ingenious and excellent remarks and cases, by Dr. W. Robertson, of Berwick, in the London Med. and Phys. Journal for November 1814, and in some subsequent Numbers, which occasioned a short and, to me, instructive correspondence between that gentleman and myself. On the same subject, too, I had the pleasure of a correspondence with Dr. Bardsley, of Manchester, and Dr. Haygarth, of Bath, in the years 1806, 7, and 10.*

The more general observations in the criticism, on the effects produced generally by digestive irritation, cannot fail to arrest the attention of those whose experience, from the accumulation of practical facts, has induced them to reflect on the subject. With respect to what is stated on the production of apoplexy, and various anomalous but greatly distressing affections of the encephalon, I have made some observations, in answer to criticisms on my pamphlet by the Eclectic Reviewer. "The connection between the diseases of the brain and a derangement of the functions of the abdominal viscera, is as conspicuous in the advanced periods of life as in our infant years. In the latter we have hydrencephalus, either rapidly hurrying the little sufferer into a premature grave, or approaching in a lingering form, with a gradually enlarging head; in the former, apoplexy of the sanguineous kind either instantly surprises the patient at the moment of apparent high health, or slowly advances in the serous kind with lethargy and carus."† The Editor of the Critical Review will there see what experience has taught me of the effects, more generally, of digestive irritation. I could here add, if time and space allowed, cases illustrative of the admirable doctrines inculcated by Mr. Abernethy, whom, as Dr. Wilson Philip justly observes, in a very excellent paper recently published, I unexpectedly met on the road of enquiry from general to local disease.‡

*King-street, St. James's-square;*
*December* 12, 1816.

---

* Dr. Yeats will be much obliged for the information, from the Editor of the Critical Review, where he can procure the Theses of Drs. Sanders and Seeds, alluded to in the criticism, as they are not published for sale.

† In the Lond. Med. and Phys. Journal published last February.

‡ Some Observations on a Species of Pulmonary Consumption. —Medico-Chirurg. Transact. vol. vii. part 2d, page 499.

